# Three Contributions to the Study of Capsicum Annuum

**Published:** 1887-01

**Authors:** 


### THREE CONTRIBUTIONS TO THE STUDY OF CAPSICUM ANNUUM.

### I. *A Brief Study of Capsicum.*

C. Carleton Smith, M. D.

It is a fact that almost every drug that has been well proven has running through it, like a scarlet thread, a peculiar symptom, which at once characterizes the drug.

The characteristic symptom which permeates Capsicum is *burning*. This is also indicative of Arsenicum, but Ars. has a peculiar and marked restlessness, which Caps. does not have.

It is also interesting to note that though this drug is used so largely on our tables as a condiment, it is, notwithstanding, one of our most precious curative agents in a potentized form.

Hahnemann tells us that a very small portion of a drop of the tincture of Capsicum diluted to the trillion-fold degree—each diluting bottle having been only twice succussed, would be found quite sufficient for a dose for all homœopathic purposes.

This drug has been of great value to me in abnormal conditions of fat, lazy people, and, perhaps, more particularly females of this description.

These patients get sick and they stick on your hands. They hang fire, so to speak, get no better and no worse. They are ill, and yet at your visits they are full of smiles. They will get out of bed for a little while, loll around the room, and then suddenly get back again. Their mouths are pasty, gums flabby, breath fœtid, with accumulations of mucus.

Give Caps. to these cases and you will frequently bring on a reaction, ending in restoration to health. And in other cases, if the remedy is not so far reaching, it will at least make the way clear for other remedies which will complete the cure.

Patients who require Caps. are generally *better* while eating, but *worse* after. They complain of their best dishes tasting sour; crave coffee and use it, but afterward suffer from attacks of dyspnœa on account of the indulgence.

It must be remembered that Caps., while it produces burning in various portions of the body, yet it has an opposite condition, and that is, icy coldness of the stomach—and, hence, the fat, lazy patient I have described, will one day complain of intense burning within the stomach, and the very next day will surprise you by reporting icy coldness in that region.

Think of this drug always in persons who lack reactive force; they want to lie down all the time; can't hold themselves up. When they walk, they totter. If they come into your office they will stagger to a chair and plump down into it with a heavy sigh, and with gasping breath describe their case.

These fat patients who require Caps. are generally plagued with hemorrhoids, which they complain of as making them feel very blue and downhearted. They are also constantly troubled with enlarged cervical glands, which are quite painful; also elongated uvulas.

The breaths of these patients are so foul that it is impossible to sit before them while they converse with you.

The stools of Caps. are somewhat like those of Nux vom. in that they are frequent and quite small; but they are accompanied by burning in return, and expelled with violence, which Nux does not have.

It also has piles with mucous discharge similar to Carbo veg., but differs from Carbo veg. in that the latter has mucous discharge of an unbearable stench, and much more profuse than Caps., even wetting the clothing through.

If a patient comes to you exceedingly gloomy from suddenly suppressed hemorrhoidal flow, give him Caps.[500] or higher.

This drug is a precious remedy for nostalgia or homesickness.

If you meet with little children who become homesick leaving their parents to go to school for the first time, give them a few doses of Caps. Young ladies going to boarding-school for the first time, will write back in a little while that they are terribly homesick. If you see them, you will find them with very red cheeks and sleepless at nights: Caps. high is the remedy.

Clumsy children who suffer with morning headache, and who have an attack of nose-bleed in the morning before they get up, require Caps.

The red cheeks of Caps. are like unto Chamomilla, but there is this difference: the latter is red and hot, the former simply red. And though feeling hot to the patient, are not so to touch. Caps. has excessive distention of the abdomen, like Lyc. The patient says her abdomen will surely burst. This does not come on, however, until about two hours after a meal—while Lac. has it immediately before the meal is finished.

This drug has also peculiar urinary symptoms. The neck of the bladder is spasmodically contracted, and the urine first comes in drops, and then in spurts, alternating.

We have marked symptoms of the genital organs which must not be overlooked—I allude to coldness of the scrotum and marked dwindling of the testicles; also violent erections in the morning when it is time to get up, which can only be allayed by cold water. We find also a gonorrhœal discharge, which is very thick and yellow. There are also pains of a rheumatic nature in the provings of this remedy, one of the most important being "bruised pain of the os-calcaneum, as if the heel were benumbed or bruised by a great leap." It also has wandering pains, like Puls., of a drawing nature in lower limbs, in back, then in upper limbs, in the nape of the neck, in the scapulæ, and in the hands; excited by moving.

Sometimes we may have to choose, in treating these fat, lazy, and dirty people, between Caps. and Sulphur. This will be your guide: the Sulph. patients dread the water, while the Caps. patients dread the air; they won't go out-doors unless you drive them out.

In summing up: Capsicum will be found most useful in patients with blue eyes and light hair. In children who are excessively clumsy, very ugly in disposition, and always complaining of being cold and chilly; persons easily offended; singing, perhaps, and full of hilarity one moment, and the next scolding furiously.

Most complaints under this remedy are aggravated by the use of coffee, more especially the cough, which latter is apt to be of an explosive character, akin to whooping-cough, so severe in its nature as to cause a sensation "as if the drums of the ears would actually burst open."

In an epidemic of diphtheria of a very severe type through which I passed in the early years of my practice, I found Capsicum of great help to me whenever it was indicated, the indications being burning and soreness of the mouth and fauces; sensation of spasmodic contraction in the throat when attempting to swallow; the throat smarts as if full of cayenne pepper. The pain in the throat is greatest, *not* during the act of swallowing, but *between*. Similar here to Ignatia, but very different in every other respect. Cheeks red, but not hot, changing to paleness, with epistaxis; chilliness between scapulæ; worse from drinking cold water.

---

### II.—*The Mind and Skin of Capsicum.*

WM. JEFFERSON GUERNSEY, M. D., PHILADELPHIA.

The mental symptom most frequently associated with Capsicum is nostalgia, and while we are not frequently called upon to prescribe for that trouble, it is so seldom written about that a brief reference to it may be of use. It has been said that "the best remedy for homesickness is to send the patient home," and while one can readily imagine some instances where this procedure would seem a necessity, it is, in a majority of cases, out of the question. We have to deal with a patient whose return home is a supposed impossibility; who not only desires to be home, but who has so dwelt upon the matter that all else has sunk into insignificance; the craving for home has become a disease, and he is, in a mild way, a monomaniac, thinking only of and wishing only for home and its attractions. Why should not this unreasonable craving call for medical treatment as well as any other?

The indication usually referred to Caps. for homesickness is

"redness of cheeks, sleeplessness, and hot feeling in the fauces." Let us also note the disposition to become angry at the least offense; the patient is peevish and irritable, or, if in a pleasant mood, will become angry from the slightest thing that disturbs them; they are inclined to be indolent and melancholy and desire to be let alone. Other remedies have been used for nostalgia, the most important ones being *Phos. ac.*, *Ignatia*, *Helleb.*, and *Bellad.*, in the order cited.

*Phos. ac.* is particularly useful to young persons who have grown too rapidly, and will suit many cases at boarding-schools. The patient is indifferent in manner, or, popularly speaking, "doesn't care whether school keeps or not;" bad effects from onanism; unwilling to talk.

*Ignatia.*—Changeable mood; sighing; distressed; "gone" feeling at stomach; not ameliorated by eating; bad effects from grief and disappointment; perhaps the patient had expected to return home on a certain date, and, being disappointed, she grieves and frets over it; or the school-girl has been reprimanded and gone to her room to cry herself sick; brooding in solitude; *imaginary* trouble; the circumstances producing homesickness not half so bad as they think.

*Helleborus.*—Irritableness, aggravated by consolation; does not want to be disturbed; weak memory; thoughtless; staring; sighing; inability to fix the mind; answers slowly, or is dull and says nothing; lamenting; moaning.

*Belladonna.*—Persons who, though naturally of jovial and entertaining dispositions, become violent, even delirious, when ill; sleepy, but cannot sleep; excitable; easily brought to tears, or morose and serious; fretful; nothing seems right; vexed at himself.

---

The skin symptoms of Capsicum are few, but we find one peculiar—hence important—symptom: namely, itching which is aggravated by scratching. I have verified this symptom several times under *Anacardium*, and, next to that remedy, we find the same symptom under *Ledum* and *Puls.* It is also recorded under *Bism.*, *Caust.*, *Con.*, *Mez.*, *Sil.*, *Stram.*, and *Sul.*, although in a minor degree.

The symptom, therefore, is more peculiar because of its unnaturalness than from the fact that it is uncommon in the *Materia Medica.* How, then, are we to tell which of these remedies to give for itching aggravated by scratching? By ascertaining what other local sensations are experienced and observing the mental idiosyncrasies.

*Anac.* and MEZER. have a change of place of the itching after scratching; *Anac.* is an ill-natured, nervous, hysterical person, with rather a malicious nature, while *Mezer.* is indifferent and despondent, and though angry at trifles, is soon sorry for it.

*Anac.* and SUL. have numbness after scratching, but *Sul.*, while nervous, peevish, and irritable, is rather inclined to be philosophical and full of religious speculations, while *Anac.* can "swear like a trooper."

*Sul.* has a raw feeling after scratching.

CAUST., PULS., *Sil.*, and *Sul.*, have sticking after scratching, but *Caust.*, while melancholy, looking on the dark side all the time, does not weep so easily as *Puls.*, and has not that slow, indecisive disposition; *Caust.* is also peevish; *Sil.* has a pretty big conscience, with compunctions about evil deeds, and though the child is obstinate and cross it cries when spoken kindly to.

CAUST., MEZ., PULS., and SUL. have stinging after scratching. Their mental states as before cited.

*Caust.* and STRONT.—Tension after scratching. STRONT. is ill-humored, inclined to be angry and impetuous, while *Caust.*, though peevish, is melancholy.

SIL. has titillation after scratching.

CAUST., *Puls.*, *Sil.*, and SUL., eruption after scratching.

*Sul.*, erysipelas after scratching.

*Caust.*, *Puls.*, and *Sul.*, papules after scratching.

*Caust.*, *Sil.*, and SUL., ulcers after scratching.

CAPSICUM has none of these indications markedly, although it has in a degree a pain in the scratched place, which is shared in by *Puls.*, *Sil.*, and SUL. This latter being the chief (as will be observed) for aggravation in general after scratching; further, *Caps.* has naturally a lackadaisical disposition not so completely found under any of the other drugs.

Thus glancing at a few of the mental and the cutaneous symptoms of this remedy, let us note that as the medicine is "peppery" so is the patient's disposition, and as we can imagine a local application of it to affect the skin, so does the internal proving of it produce burning and an itching, the latter not relieved by scratching any more than would an ordinary rubbing remove the dust of pepper from the skin.

I venture the assertion that in no class of diseases are mental symptoms less noted than in skin troubles, yet where will we find a diseased skin (cutaneous manifestation of constitutional disturbance) free from mental symptoms? We expect our patients to be anxious about these troubles, but sometimes they are unnecessarily so, thus aiding us in the selection of a remedy; or if indifferent, it is still stranger and of greater importance. Their pride may be deeply wounded, and they avoid society from mortification because of a small, almost unnoticeable, eruption. All of these points are noteworthy, and if taken into account will aid us in selecting the truly homœopathic simillimum without which there is no perfect cure.

---

### III.—*A Few Comparisons of Capsicum.*

JOHN V. ALLEN, M. D., PHILADELPHIA.

I have endeavored, gentlemen, to give a few comparisons of the medicines having somewhat similar symptoms to Capsicum, the subject of the paper just read by Dr. Smith, and selected a few symptoms from each of the following headings: eye, ear, vomiting, stool, abdomen, cough, and chill.

Under the eye symptoms of Hering's *Mat. Med.*, we find objects appear black. It is the only remedy that I have been able to find having this symptom. *Phos.* has objects appear dark, as also have Bell. and Hep., while Thuja has objects appear dark while reading. The dim vision of Caps., particularly in the early morning, might be compared with Cham., Chelid., and Puls., each of which have it, with the difference that Caps. is better on rubbing the eyes, and the totality of symptoms in a given case will decide which one of the other three might be indicated.

The ear symptom of Caps., I think, should not be hastily overlooked, as it produces one condition which is so often maltreated with the knife, and very often proves fatal to the patient, and that is caries of mastoid process, which may be on either side, and here you must compare with Aurum. The Caps. swelling and inflammation is very tender to touch, with smarting, burning, tearing pains, while in Aurum. we have the mercurio-syphilitic cachexia, generally with boring pains, offensive otorrhœa, and a general breaking down of the osseous system. Each of these remedies has pàin behind the left ear. The Caps. pain is tearing in character, while that of Aurum is characteristically boring. Not only do we find the adjacent parts of the ear affected as I have shown, but we have an aching in one or both ears when coughing. This symptom I consider peculiar, and you will find not only the ear but any distant part of the body affected by coughing. You must compare Dioscor. with Caps. in pain affecting the ears on coughing, which in Dios. is worse at eight A. M. I might here mention that Hep. and

Phos. ac. each have earache when blowing the nose, and Manganese from laughing.

The eructations of Caps. are peculiar, and the patient will tell you the drug has the taste of the gases raised. I might mention that it has stitches in the side when belching. Now, Sepia should be compared when this symptom is found, and other symptoms of Sep. will lead you to select it.

In Sepia I might again mention that we have relief of pains in the back from eructations, and in Ars. pains in the back on belching. Verbascum should not be forgotten for belching during cutting pains in the abdomen. I will now try to compare the abdominal and stool symptoms together, but will not go into the finer shades of symptoms, but only mention comparative remedies.

In colic, around the umbilicus, with mucous stools, sometimes streaked with blood, with tenesmus, we must compare Merc., Canth., Coloc.

In Merc. you have the never-get-done feeling and chilliness following every stool, whereas in Caps. the chilliness is not after stool but after drinking, and violent thirst follows every stool, and must go to stool immediately after drinking, passing nothing but mucus, with burning in the rectum and bladder; it is with this latter symptom that we should think especially of Canth., as it has slimy, bloody stool, with burning pains in the rectum and anus, causing the patient to cry out; it also has chilliness after stool, as if cold water were poured over the body, and more tenesmus of the bladder than its ally Caps., and its vesical symptoms are always present when indicated, and we should not forget Merc. cor. when vesical symptoms are present in this character of diarrhœa. To Colocynth. I will give a passing notice, in merely stating that the tenesmus is during stool and relief of pain after stool, and the colic, as you know, is relieved by bending double and hard pressure, which is not so in Caps. Thrombidium should not be overlooked with symptoms presented like the above mentioned. It has, like Caps., mucous, blood-streaked stools, thirst following every stool, tenesmus, tenesmus and chills in the back, but the Thromb. patients cannot wait until they are through eating, but have the passage while eating (like Ferrum). Before leaving this section I would like to call your attention to Caps. in hemorrhoids, and its relation to other drugs in this condition. It has hemorrhoids which are burning, swollen, itching, throbbing, with sore feeling in the anus; bleeding or blind, with mucous discharge. Mur.-ac. should be one of the first remedies thought of when the sore feeling of the anus is complained of, as it is one of the few remedies having hemorrhoids which are too sore to bear the least touch. Sulph. and Baryta-carb. should be carefully studied when mucous discharge accompanies piles, and the distinctive characteristic symptom of each will differentiate them from Caps. The cough of this remedy, as I have said before, causes pain in distant parts, as aching in the ears, nosebleed, stitches in hypochondria, stitches in neck of bladder, and stitches and tearing from hip to knee and foot, but you will find one peculiar cough symptom which I will mention and compare with Sang., and that is with every expulsive cough (and at no other time) there escapes a volume of pungent, fetid air. The Sanguinaria cough has belching before and after the cough, and only after in Caps., and the Sang. breath and sputa smell badly, even to the patient, and the cough is relieved by passing flatus up and down. Caps. sputa is dirty brown and not offensive to patient.

The grandest sphere of the action of Caps. is its power to cure intermittent fever, and its indications are peculiar and not difficult to differentiate. The chill begins in the back, with thirst, worse after drinking, better when walking in the open air.

Eupat. per. and purp., Lach., and Polyporus have each chill, commencing in the back.

Eupat. per. has insatiable thirst, but drinking causes nausea and vomiting and hastens chill, thirst two to three hours before chill, and chill ends in bitter vomit.

Lach. chill, as also does *Eupat. purp.*, commences in the small of the back, and not between the shoulders, as Caps. and Polyp.; patient wants to be near fire, while Caps. is better walking in the open air; Lach. has no thirst during chill, as do Caps. and Eup. per.

Polyp. has chill commencing in back between shoulder-blades, like Caps., but is worse while in the open air.

The Caps. chill, which is often followed by sweat, without intervening heat, should be compared with Causticum and Lyc. Caust. chill is lessened by drinking, and is without thirst, followed by sweat, without intervening heat.

Lyc. chill generally commences at four P. M.; no heat after chill, but great thirst after the sweat.

During the Caps. chill we sometimes have vomiting of phlegm. In this it should be compared with Ign. and Puls. The heat of Caps., which is lessened by motion, should be compared with Ferrum.

Characteristically all the stages of Caps., *viz.:* chill, fever, and sweat, are lessened by motion, and the chill spreads generally until extreme points are reached, then as gradually declines.

